# In Vitro Consequences of Electronic-Cigarette Flavoring Exposure on the Immature Lung

**DOI:** 10.3390/ijerph16193635

**Published:** 2019-09-27

**Authors:** Sara K. Berkelhamer, Justin M. Helman, Sylvia F. Gugino, Noel J. Leigh, Satyan Lakshminrusimha, Maciej L. Goniewicz

**Affiliations:** 1 Department of Pediatrics, University at Buffalo, SUNY, Buffalo, NY 14203, USA; 2 Department of Health Behavior, Roswell Park Cancer Institute, Buffalo, NY 14263, USA; 3 Department of Pediatrics, University of California Davis, Davis, CA 95616, USA

**Keywords:** electronic cigarettes, electronic cigarette flavorings, toxicity, immature lung, lung development

## Abstract

*Background:* The developing lung is uniquely susceptible and may be at increased risk of injury with exposure to e-cigarette constituents. We hypothesize that cellular toxicity and airway and vascular responses with exposure to flavored refill solutions may be altered in the immature lung. *Methods:* Fetal, neonatal, and adult ovine pulmonary artery smooth muscle cells (PASMC) were exposed to popular flavored nicotine-free e-cigarette refill solutions (menthol, strawberry, tobacco, and vanilla) and unflavored solvents: propylene glycol (PG) or vegetable glycerin (VG). Viability was assessed by lactate dehydrogenase assay. Brochodilation and vasoreactivity were determined on isolated ovine bronchial rings (BR) and pulmonary arteries (PA). *Results:* Neither PG or VG impacted viability of immature or adult cells; however, exposure to menthol and strawberry flavored solutions increased cell death. Neonatal cells were uniquely susceptible to menthol flavoring-induced toxicity, and all four flavorings demonstrated lower lethal doses (LD50) in immature PASMC. Exposure to flavored solutions induced bronchodilation of neonatal BR, while only menthol induced airway relaxation in adults. In contrast, PG/VG and flavored solutions did not impact vasoreactivity with the exception of menthol-induced relaxation of adult PAs. *Conclusion:* The immature lung is uniquely susceptible to cellular toxicity and altered airway responses with exposure to common flavored e-cigarette solutions.

## 1. Introduction

Electronic nicotine delivery devices (ENDS), or electronic cigarettes, have been developed as a safer and less toxic substitute to smoking. Use of these products is growing rapidly, and predictions based on current sales suggest that consumption of e-cigarettes could surpass that of conventional cigarettes within the next decade with estimates of a $50 billion global market by 2025 [1,2]. While established smokers can avoid known tobacco toxicities with e-cigarette use, vulnerable populations may exist who are more susceptible to exposure. Pregnant women as well as parents and caregivers of infants may rationalize a switch to these products to reduce risks for children. Indeed, studies have shown that e-cigarette use is increasing sharply among women of reproductive age and that they are perceived by many as safer alternatives during pregnancy [3,4,5,6]. An online survey of 455 pregnant women recruited from a national website survey service (Amazon Mechanical Turk) identified higher rates of vaping as compared to smoking with 6.5% of respondents reporting sole use of e-cigarettes, 5.6% reporting sole use of conventional cigarettes, and an additional 8.4% reporting use of both products [7]. A clinical survey performed at a university-based clinic (Little Rock, Arkansas) implied even higher rates in an obstetrical cohort with 11.9% reporting current e-cigarette use. [8]. Oncken et al. similarly found that 14% of participants in a smoking cessation trial reported use of e-cigarettes during pregnancy, most commonly to quit [9]. Despite these perceptions and practices, the impact of exposure to e-cigarettes on the fetal and the newborn population during this vulnerable period of development remains unknown. Clinical studies to investigate effects of in utero and postnatal secondhand exposures are challenged by highly variable products and patterns of use, complex ethics, and issues with compliance.

While data from clinical studies and animal models suggest e-cigarettes reduce risks of adult tobacco-related disease [10,11], concerns remain for their impact with fetal or neonatal exposures [12,13,14]. Although the consequences of pre and postnatal nicotine exposure have been characterized (including concerns for compromised pulmonary development and lung function as well as adverse neurocognitive and behavioral outcomes [15,16,17,18,19,20,21]), little is known regarding the impact of early exposure to the numerous additional constituents of e-cigarette refill solutions, including propylene glycol (PG), vegetable glycerin (VG), and commonly used flavorings. Studies in undifferentiated embryonic cells raised concerns for increased susceptibility to cell death with exposure to e-cigarette solutions [22]. Specifically, nicotine and flavored e-cigarette refill solutions induced greater cytotoxicity in undifferentiated human embryonic stem cells (hESC) as well as mouse neural stem cells (mNSC) as compared to more mature human pulmonary fibroblasts (hPF).

Indeed, the unique characteristics of the immature lung may render the fetus and the newborn more vulnerable to injury with exposure to toxicants. Compromised antioxidant defenses and altered inflammatory responses can contribute to age-dependent susceptibilities [23,24,25]. As an example, we previously demonstrated that supraphysiologic oxygen induces exaggerated mitochondrial oxidant stress as well as increased cell death in neonatal as compared to adult lung cells [26]. In addition, compromise of alveolar and vascular development observed with neonatal hyperoxia is developmentally regulated with greater evidence of lung injury induced by early as opposed to late exposure [27].

Specific attributes of fetal and newborn physiology further influence susceptibility to chemical exposures [28]. Increased respiratory rates in newborns can increase environmental exposure to secondhand aerosols. Lower metabolic rates, variable enzymatic activity, decreased concentration of plasma binding proteins, as well as reduced renal and biliary excretion can all further impact chemical exposures in a fetus or a newborn. A higher percentage of water versus body fat in the fetus and the newborn provide opportunity for lipophilic substances to accumulate while water soluble compounds can also concentrate in utero, as these chemicals are commonly excreted into amniotic fluid with reabsorption through the immature fetal skin. As a prime example, higher nicotine levels have been observed in fetal tissue as compared to maternal blood [29]. Collectively, these physiologic and cellular mechanisms impact bioavailability and may exaggerate risks of toxicity with fetal or neonatal exposures to the chemical constituents present in e-cigarettes.

Despite the common presence of added flavoring chemicals in e-cigarette products, little is known regarding the safety and the long-term health effects associated with aerosolized exposure to these constituents [30,31,32]. The concentrations of flavor chemicals in e-cigarette fluids are sufficiently high for toxicity to be of concern both for adults who use these products and for children who may be exposed in utero or via secondhand inhalation. Studies have documented that flavorings make up more than 1–4% (10–40 mg/mL) of e-cigarette refill solution volume, whereas nicotine typically ranges from 0.6–2.4% (6 to 24 mg/mL) [33]. Online vaping forums suggest that use of 5 mL per day of flavored e-liquids would be common, resulting in significant cumulative exposure with chronic use [34]. While the extent to which these chemicals are delivered to the fetus and the newborn remains unknown, in utero exposures are a valid concern, with a murine model identifying alterations in the hippocampus of offspring exposed to nicotine-free flavored e-cigarettes during gestation [14]. Additionally, flavoring chemicals that are not effectively retained in the user’s lungs would be exhaled by e-cigarette users, leading to concerns for secondhand exposure of newborn and infants in proximity of caregivers who vape [35,36,37].

Flavoring solutions commonly contain a mixture of numerous chemicals; as an example, analysis of 28 representative e-liquids identified over 140 different flavoring chemicals present in one published panel [38]. While many of these chemicals are commonly ingested with food products and have been certified as “Generally Recognized as Safe” (GRAS) by the Flavoring and Extracts Manufacturers Association (FEMA), this designation is in reference to safety with ingestion or topical exposure only and would not apply to inhalation. Indeed, the bioavailability and the consequence of exposure with vaping of flavoring chemicals deemed as safe for ingestion remain unknown.

With growing appreciation for toxicity of flavoring chemicals [30,32,39,40,41,42] and a specific concern for the consequences of exposure on the vulnerable developing lung, we sought to determine the relative toxicity and the impact on physiologic responses in immature lungs cells and tissue exposed to popular nicotine-free e-cigarette flavored solutions [34,43]. Unique access to fetal, newborn, and adult ovine pulmonary cells lines as well isolated neonatal and adult ovine lung tissue allowed evaluation of developmental susceptibility. We hypothesized that the fetal and the neonatal lung would be more vulnerable to flavoring solution-induced toxicities and that airway and pulmonary vascular response with exposure may also be developmentally regulated.

## 2. Materials and Methods

### 2.1. Flavored E-Cigarette Refill Solutions

Convenience samples of flavored solutions used for these studies were labeled as made by Ecto World (https://www.ectoworld.com) and purchased locally from a single vender (Cloud 9 Smokes and Vapors, 476 Elmwood Ave, Buffalo, NY, USA). Products were labeled as Menthol, Vanilla Cupcake, American Tobacco, and Strawberry Blast with 0 mg nicotine. These four were selected as a pilot to both assess actual products in use and to include a range of popular flavorings based on published literature [34,38,43]. Non-targeted analysis of e-liquids was performed as previously published using gas chromatography–mass spectrometry (GCMS) to determine constituents and confirm that samples were nicotine free [30]. Briefly, 10 µL of each sample was diluted with 1 mL methanol (Fisher Scientific; Waltham, MA) and analyzed by GCMS using an Agilent 7890B GC and an Agilent 5977A MS (Santa Clara, CA). An HP−5 30 m × 0.320 mm × 0.25 m capillary column (Agilent) with a flow rate of helium of 1.7 mL/min was utilized. Temperature of the injector and the detector was 250 °C, and the column temperature increased from 110 to 250 °C (10 °C/min) with a hold for 1 min. The injection volume was 1 µL with a split ratio of 40:1. Qualitative analysis of the flavored liquids was carried out using the NIST 14 Mass Spectral Library (U.S. Department of Commerce) as well as the Flavors and Fragrances of Natural and Synthetic Compounds 3 flavoring library.

### 2.2. Cell Culture

Primary pulmonary artery smooth muscle cell (PASMC) cultures were prepared from intrapulmonary arteries collected from healthy 125 (gestational) day fetal, 2 day newborn lambs, and adult ewes as previously published [44]. Numerous publications support our use of this large animal model, as ovine pulmonary development and physiology parallel human, while cellular metabolism and signaling in isolated tissue and cell lines correlate with findings in human disease [44,45,46,47,48,49,50,51,52,53]. For toxicity studies, cells were maintained in Dulbecco’s Modified Eagle Medium (DMEM) with 10% fetal bovine serum (FBS), 2 mM l-glutamine and 100 units/mL penicillin, 100 mg/mL streptomycin (P/S) under standard cell culture conditions (37 °C with 5% CO_2_). All experiments were carried out between passages 3 and 8. Cells were seeded onto 12 well plates at approximately 8000 cells per cm^2^ and grown to 60–70% confluency. Fetal, newborn, and adult PASMC were subsequently synchronized by transfer to serum-free DMEM + P/S for one hour prior to treatment in parallel with select nicotine-free flavored solutions, pure propylene glycol (PG), or pure vegetable glycerin (VG) (Fisher Scientific, NH) at 1:100, 1:1000, or 1:10,000 dilutions. Cell death assays were performed following 24 h incubation.

### 2.3. Cell Death

Cell death was determined using a lactate dehydrogenase (LDH) assay (Biovision, San Francisco, CA). Using an enzymatic coupling reaction, LDH was quantified in the supernatant and the tritonized cell lysate. LDH activity was determined by spectrophotometric reading at OD_450_. Percent cell death was calculated as concentration in supernatant/concentration in lysate × 100. Dilution at which 50% cell death would occur (lethal dose or LD50) was calculated assuming linear dose dependency (from studies in which testing of two doses concurrently was possible) with extrapolation of values out to a 1:10 dilution.

### 2.4. Isolated Airway and Vessel Studies

Airways and vessels from pregnant ewe and newborn lambs were collected following sacrifice of animals utilized for a separate experimental protocol evaluating transitions at birth. For those studies, newborn lambs were delivered by c-section as part of an Institutional Animal Care and Use Committee at the State University of New York at Buffalo approved protocol (PED10085N). C-section was performed under 4% isoflurane anesthesia. Newborn lambs were ventilated for 4–6 h prior to sacrifice, during which time they received fentanyl titrated from 0–5 mcg/kg/h. Ewes and lambs were euthanized following delivery and completion of the study with pentobarbital (100 mg/kg). Animals were exposed to purified air or oxygen only prior to sacrifice.

The intrapulmonary arteries (PA) and the fifth generation bronchial rings (BR) were dissected, washed in Krebs solution (NaCl 119 mM, KCl 5.4 mM, CaCl_2_ 2.5 mM, KH_2_PO_4_ 0.6 mM, MgSO_4_ 1.2 mM, NaHCO_3_ 25 mM, and glucose 11.7 mM), and hung in vessel baths aerated with 21% O_2_ and 6% CO_2_ to achieve a pH of 7.4, as described previously [50]. The samples were mounted and stretched to a passive tension of 800 mg (PA) and 1000 mg (BR) to optimize reactivity to experimental agents. A continuous recording of isometric force generation was obtained by tying each vessel ring to a force displacement transducer (model UC2, Statham Instruments, NY) that was connected to a recorder (Gould Instrument, OH). PA were pretreated with propranolol (10^−6^ M) to block the beta adrenergic effects and constricted with increasing titrations (10^−8^ to 10^−5^ M) of norepinephrine (propranolol and NE, Sigma Aldrich, MO). BR were constricted with increasing titrations (10^−8^ to 10^−5^ M) of 5-hydroxytryptamine (5-HT, Sigma Aldrich, MO).

To study responses to flavored solutions, PA and BR were preconstricted with half maximal effective concentration (EC50) of NE or 5-HT followed by treatment with a flavoring or 50/50 PG/VG at a 1:1000 dilution with companion preparation (5-HT or NE) alone used as time controls. Preconstriction to EC50 allowed tested e-liquids to further constrict or relax as is physiologically relevant. Following experimental treatment, PA and BR were washed with Krebs solution. Maximal contractile response to 118 mM/L of potassium chloride (KCl) was obtained. Wet tissue weights were obtained at the end of each experiment, and constriction responses were normalized to tissue weight and compared as percentages of relaxation of the EC50 constriction. Data were analyzed as force of constriction per unit of tissue weight (grams of force per gram of tissue weight) of the vasoconstrictor agent. Response to flavored solutions was expressed as a percentage of maximal constriction of 5-HT or NE.

### 2.5. Statistical Analysis

Continuous variables were analyzed using a Student’s t-test or ANOVA as appropriate with Tukey’s post hoc analysis. A *p* value of ≤0.05 was considered statistically significant. For cellular experiments, 2–3 wells on a plate were treated for each condition, and the average result considered one n with >6 n from independent cell platings for all studies. For vessel and airway reactivity, replicate tissues from 4 adult and 6 neonatal lambs were studied.

## 3. Results

### 3.1. Chemical Analysis of Flavored Liquid Solutions

Non-targeted GCMS analysis confirmed that the Ecto flavored solutions did not include nicotine in two batches of the four flavors used in the study. The two batches were purchased nine months apart with the second batch being nine months old at chemical analysis. Consistent with prior publications [33,38], non-targeted GCMS analysis identified a large number of chemicals present in each of the four samples with variability between batches (Table 1).

**Figure 1:**
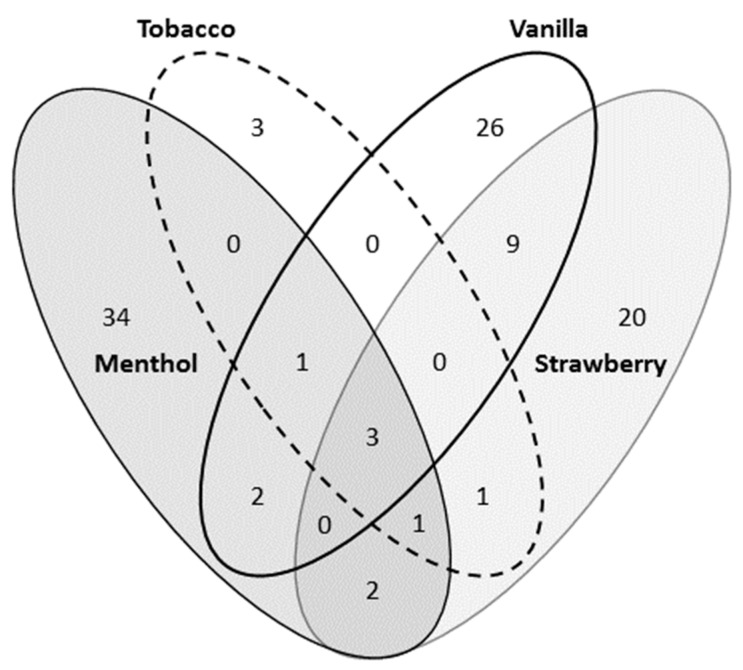
Flavoring composition of four popular flavored refill solutions. GCMS performed on flavored solutions identified notable overlap of flavoring chemicals present with three present in all four analyzed and an additional 16 present in two or more. Analysis also identified a wide range in the number of unique flavorings present from three in tobacco to 34 in menthol. See the Supplemental Table S1 for specific chemicals.

Flavorings common to all four included benzophenone (CAS 119-61-9), hydroxyacetone (CAS 116-09-6), and ethyl maltol (CAS 11-8-4940). Unique flavoring chemicals were identified with each flavored solution; however, there was significant overlap in chemicals present in the four studied. Menthol had the most unique chemical flavorings present while tobacco had the least (Figure 1 and Supplementary Table S1). GCMS did not allow for quantification of the specific chemicals present.

### 3.2. Cell Toxicity Studies

**Figure 2:**
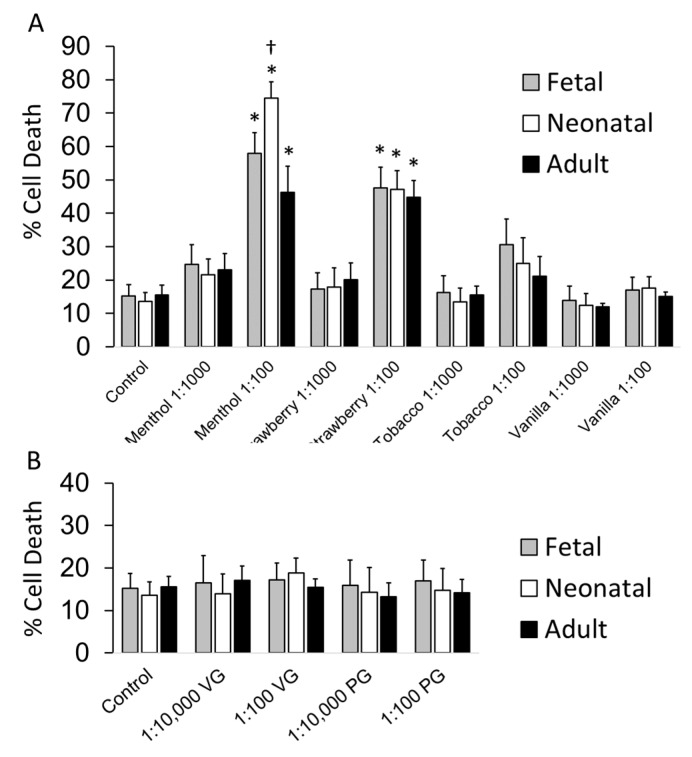
Exposure to select flavored solutions induced cell death in pulmonary artery smooth muscle cells (PASMC). PASMC isolated from fetal, neonatal, and adult ovine lungs were treated with variable concentrations of flavored solutions or pure vegetable glycerin (VG)/propylene glycol (PG) for 24 h. (**A**) The 1:100 dilution of menthol and strawberry resulted in significant increase in cell death as compared to untreated controls (* *p* < 0.05). Immature cells were more susceptible to menthol toxicities († *p* < 0.05 as compared to adult). (**B**) The 1:100 and the 1:10,000 VG and PG exposure did not result in significant increase in cell death. Data are presented as mean + SEM. *n* ≥ 6.

As pulmonary blood vessels actively promote alveolar development and contribute to the maintenance of alveolar structures throughout postnatal life [54], we chose to evaluate the toxicity of flavored solutions on pulmonary artery smooth muscle cells (PASMC). To determine relative toxicity in immature versus mature cells, fetal, neonatal, and adult PASMCs were exposed to a select panel of flavored solutions, pure PG, or VG for 24 h at variable dilutions (1:100, 1:1000, 1:10,000). Cell death was flavoring dependent, with the greatest to the least toxicity observed with menthol, strawberry, tobacco, and vanilla flavorings. Exposure to 1:100 dilution of menthol and strawberry solutions induced significant cell death in fetal, neonatal, and adult cells (control: 15.2 ± 3.5, 13.6 ± 2.7, 15.6 ± 2.9%; menthol: 57.9 ± 6.2, 74.5 ± 4.9, 46.3 ± 7.7%; strawberry: 47.6 ± 6.2, 47.2 ± 5.5, 44.8 ± 7.9% for fetal, neonatal and adult PASMC, respectively). Immature cells were more susceptible to menthol toxicity with the greatest cell death documented in neonatal cells (Figure 2A). In contrast, exposure to tobacco and vanilla solutions did not significantly impact viability as compared to untreated controls. In addition, treatment with unflavored, nicotine-free VG and PG resulted in no significant increase in cell death (Figure 2B), implicating additional flavoring or non-flavoring chemicals present in the refill solutions for the death observed in Figure 2A. Calculated LD50s suggested increased susceptibility to flavored solutions in immature PASMC as compared to adults, with lower calculated concentration noted for fetal and neonatal PASMCs in all four tested flavorings as compared to adults (Table 2).

### 3.3. Airway and Vessel Reactivity Studies

**Figure 3:**
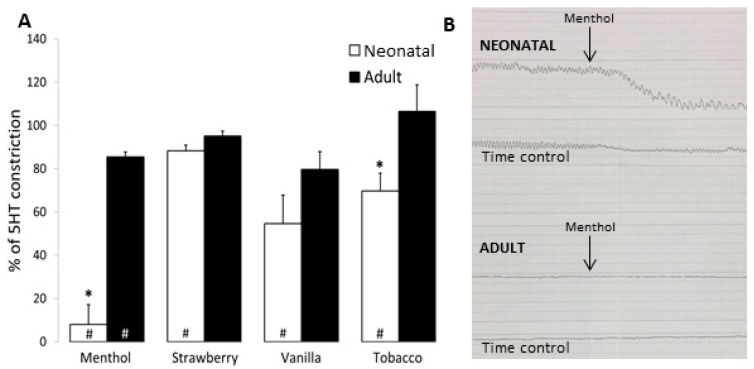
Flavored solutions induced greater bronchodilation in newborn as compared to adult bronchial rings. Bronchial rings (BR) isolated from neonatal and adult ovine lung were treated with flavored solutions at 1:1000 dilution. (**A**) Menthol, strawberry, vanilla, and tobacco flavorings all induced bronchodilation of neonatal BR, while only menthol induced bronchodilation in adults (# *p* < 0.05 as compared to time control). Neonatal airway demonstrated significant decrease as compared to adult with both menthol and tobacco exposure (* *p* < 0.05 as compared to adult). Data are presented as mean + SEM. *n* = 3–8. (**B**) Representative tracing demonstrating neonatal and adult responses in BR treated with menthol flavoring (top) as compared to untreated (time control).

*Bronchial Rings:* To determine whether airway response to flavored solutions was also developmentally regulated, both neonatal and adult intrapulmonary bronchial rings (BR) were preconstricted with EC50 doses of 5-HT. Once plateau contraction was achieved, BR were exposed to nicotine-free, unflavored 50%/50% PG/VG as well as selected flavored solutions at 1:1000 dilution. Treatment with PG/VG did not impact EC50 constriction of neonatal or adult BR (92.6 ± 3.5% and 98.4 ± 5.1% constriction of time control, respectively). Treatments with 1:1000 dilution of menthol, strawberry, vanilla, and tobacco e-liquids all resulted in significant airway relaxation in neonatal BR as compared to untreated time controls (*menthol:* 8.0 ± 9.1; *strawberry*: 88.2 ± 2.8; *vanilla*: 54.6 ± 13.2; *tobacco*: 69.7 ± 8.2% constriction of neonatal time control) (Figure 3A). In contrast, exposure to the same concentrations of 1:1000 dilution of strawberry, vanilla, and tobacco did not impact airway relaxation in adult BRs, although some relaxation with menthol was observed (*menthol:* 85.5 ± 2.2% constriction of adult time control). Neonatal airway bronchodilation with exposure to menthol and tobacco flavored solutions was significantly greater than that observed in adult BR, with marked bronchodilation noted with menthol (Figure 3B).

Contraction to EC_50_ 5-HT elicited a force of 103 ± 14 g/g with a maximum constriction with KCl of 171 ± 23 g/g in adult BRs. Similarly, neonatal constriction to 5-HT was 54 ± 12 g/g with a maximum constriction with KCl of 98 ± 19 g/g. These data support comparable contractions, as 5-HT elicited 60% and 55% of max contraction for neonatal and adult BR, respectively.

**Figure 4:**
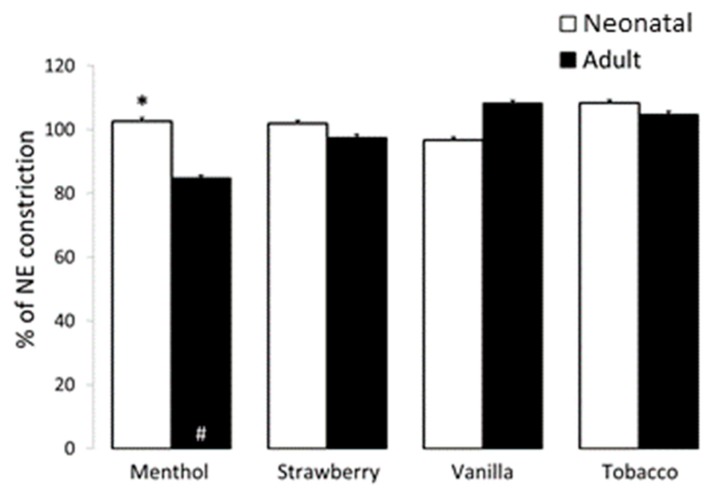
Effect of flavored solutions on vasodilation in newborn and adult pulmonary arteries (PA). The 1:1000 dilution of strawberry, vanilla, and tobacco flavored solutions did not impact vascular constriction in neonatal or adult PA. However, exposure to 1:1000 dilution of menthol flavoring resulted in significant relaxation of adult but not neonatal PA (# *p* < 0.05 as compared to time control, * *p* < 0.05 as compared to adult PA). Data are presented as mean + SEM. *n* = 3–8.

*Pulmonary Arteries:* To evaluate pulmonary vascular response to flavored refill solutions, neonatal and adult pulmonary arteries (PA) were preconstricted to EC50 with NE. Once stable constriction was obtained, PA were similarly treated with 1:1000 nicotine-free, unflavored 50%/50% PG/VG and selected flavored solutions. The 1:1000 dilution of PG/VG did not impact vasoconstriction in either the neonate or the adult (101.4 ± 6.6 and 98.9 ± 2.4% constriction for neonate and adult, respectively). While comparable dosing of flavored solutions resulted in relaxation of bronchial rings, no significant change was noted with exposure of neonatal or adult PA to 1:1000 dilution of strawberry, vanilla, or tobacco. However, treatment with 1:1000 dilution of menthol induced relaxation of adult but not neonatal PA (85.5 ± 2.2 and 102.6 ± 3.2% constriction of time control for adult and neonate) (Figure 4).

Contraction to NE after propranolol elicited a force of 512 ± 6 g/g with a maximum constriction with KCl of 538 ± 53 g/g in adult PAs. Similarly, neonatal constriction to NE was 126 ± 37 with a maximum constriction with KCl of 148 ± 49 g/g. These data support comparable contractions, as NE elicited 95% and 85% of max contraction for neonatal and adult PA, respectively.

## 4. Discussion

Our data suggest that the fetal and the neonatal lung may be at increased susceptibility to toxicity with exposure to flavored e-cigarette solutions and that physiologic responses to these common additives may also be altered in the immature lung. While flavored solutions have previously been shown to be harmful to lung epithelial cells in some but not all in vitro models [30,31,32,55], these studies are the first to identify a developmental susceptibility with exposure of differentiated lung cells. Further, our results are novel in demonstrating that physiologic response to e-cigarette flavoring solutions may also be developmentally regulated.

GCMS analysis identified a large number of non-flavoring and flavoring chemicals present within each of the solutions with notable differences in products purchased nine months apart. While these differences may in part be attributed to aging and chemical decomposition, our data more likely represent the variability within products produced by a specific vendor [22]. Batch variability has been well described and may account for some of the range in responses that we saw in our experiments as well.

We further noted a wide range of toxicity with exposure to a limited panel of flavored solutions. Both menthol and strawberry were noted to be highly toxic, consistent with prior publications [30,56]. While Bahl et al. suggested that the number of peaks on high-performance liquid chromatography (HPLC) correlate with cytotoxicity [22], we noted a comparable number of total and flavoring chemicals (by GCMS) in both toxic and non-toxic products. Importantly, as no nicotine was detected in all tested products, the observed findings can be attributed to the added chemicals in flavoring solutions.

Of interest, while only two of four flavorings demonstrated significant toxicity at a 1:100 dilution, all four flavorings induced bronchodilation in neonatal bronchial rings at a 1:1000 dilution with limited or no impact on adult airway constriction at the same dose. As menthol has previously been shown to inhibit airway smooth muscle contraction in tracheal tissue isolated from guinea pigs and rats [57,58], we suspected all four flavoring samples might include some quantity of menthol or one of its chemical derivatives. However, GCMS did not confirm this suspicion with identification of (±)-menthol, (±)-menthone, (-)-menthone only in the product referred to as menthol, indicating that additional flavoring chemicals are capable of inducing relaxation of neonatal airways.

Bronchodilation in the neonatal airways suggests that newborns and infants in proximity to secondhand flavored e-cigarette vapors may be at risk of exaggerated exposure to a higher concentration of these and additional aerosolized chemicals (including nicotine when present) via increased delivery to the alveoli. Increased minute ventilation in infants would further facilitate the delivery of these aerosolized chemicals, compounding concerns for risk of toxicity in the immature lung.

Vaporized e-cigarette solutions and flavoring chemicals have been shown to contain notable quantities of potentially toxic reactive oxygen species (ROS) [32,59]. Chemicals present in flavored refill solutions may also be capable of inducing intracellular oxidative stress with absorption. The developmental susceptibility to flavoring-induced toxicity and bronchodilation that we observed in these studies may reflect compromised antioxidant capacities present in the immature lung [23], as both cell death and airway responses are mediated by ROS [60,61,62]. Alternatively, altered cellular metabolism, variable cellular absorption, or a difference in the immature inflammatory response [25] could contribute to developmentally regulated responses.

While 1:1000 dilution of each of our studied e-liquids elicited bronchodilation in the newborn airway, this dilution did not impact the pulmonary vasculature with the exception of vasodilation of adult pulmonary arteries with exposure to menthol flavored solutions. Menthol is a common and highly popular flavoring with both vaping and conventional cigarettes, with surveys implying that 27% of smokers report use of mentholated products [63]. Pulmonary vasodilation (as observed in the adult PAs) with menthol inhalation could facilitate absorption of e-cigarette constituents including nicotine, raising concern for potential exacerbation of toxicities and increased gestational exposure. Indeed, some studies suggest that mentholated products may be associated with increased pulmonary pathology [64,65].

While our data are novel in evaluating the consequences of exposure in fetal and neonatal pulmonary cells as well as immature lung tissue, there are several limitations to these studies. Notably, e-cigarette refill solutions were added directly to cell cultures or tissue perfusate, resulting in an exposure that failed to include toxic products generated with heating and aerosolization, avoided the process of thermal degradation, and was potentially at an inaccurate pH [66]. While analytical evaluation demonstrated highly efficient transfer of flavoring chemicals to aerosol (mean transfer 98% at both 3V and 5V) [67], unique chemicals produced with vaping of flavored refill solutions are not represented in these studies. Further, variable temperatures, voltage, devices, and puffing patters have all been shown to modify aerosol toxicity and were not explored [68]. We acknowledge that these limitations impact the interpretation of these studies and fail to meet optimal modeling suggested by Behrsing et al. [69]. We utilized direct exposure with the technical challenges of delivering aerosolized product into the tissue perfusate and to cultured PASMCs.

In addition, our study does not distinguish the effect of specific flavoring chemicals or eliminate the potential contribution of impurities limiting our conclusions. Future studies investigating the specific chemicals of interest individually with known purity and concentrations present in refill solutions would be indicated. We also acknowledge that our small panel fails to characterize the extensive library of now over 7000 unique flavors on the market and may not be generalizable to other e-cigarette products [70]. Finally, the toxicity of the flavoring solutions was not contextualized with comparison to tobacco; ultimately, these products may represent a safer alternative to smoking.

We are further limited by the lack of data regarding typical concentration of flavoring chemicals that lung cells, pulmonary tissue, and a fetus might be exposed to. Online vaping forums suggest that use of 5 mL per day of flavored refill solutions would be common [33]. Our dosing range maxed at 1% to model some degree of dilution and was based on rationale provided in other publications [56]. Specifically, based on particle size, the predicted deposition of e-cigarette aerosol in the lungs is 15–45% [71]. Given that the airway surface liquid volume in the lung is approximately 3 mL [56], vaping of 10 mL would lead to a dilution of 33–60%, suggesting that our experimental dosing of 0.1–1% may be in reasonable range for PASMC and tissue exposure. While the specific concentrations with fetal exposure remain unknown, recent publications have demonstrated that flavoring chemicals can indeed impact fetal development [14].

Finally, while these studies are novel in comparing toxicity and physiologic responses with exposure in both neonatal and adult models, responses were limited to isolated cells and tissue. Our data suggesting a developmental susceptibility to commonly used flavored solutions advocate for further evaluation of these chemicals by way of *in vivo* exposure to the aerosolized product.

## 5. Conclusions

Gestational and postnatal exposure to electronic cigarettes represents rapidly growing threat to the fetal and the newborn population. Use of added flavorings with vaping has far out-paced our understanding of their implications for public health. Our data suggesting a developmental susceptibility with exposure to common flavoring solutions argue the critical need for further thoughtful evaluation of these products.

## Figures and Tables

**Table 1:** Chemical analysis by gas chromatography–mass spectrometry (GCMS). GCMS was performed on two batches of Ecto flavoring solutions, confirming absence of nicotine but chemical variability with differences in the number of chemicals present in batches of the same flavored solution.

	Menthol	Strawberry	Tobacco	Vanilla
***All chemicals***				
Batch 1	76	83	37	77
Batch 2	72	72	39	66
Propylene glycol (PG)	Positive	Positive	Positive	Positive
Vegetable glycerin (VG)	Positive	Positive	Positive	Positive
Nicotine	Negative	Negative	Negative	Negative
***Flavoring chemicals***				
Batch 1	44	39	12	43
Batch 2	35	34	14	34
% flavorings	53%	47%	34%	54%
***Major flavoring*** ***chemicals detected***	(±)-Menthol Pulegone Alpha-pinene Anethol (E)-Cinnamyl acetate Linalool	Methyl cinnamate Benzyaldehyde Isovaleraldehyde Linalool	Beta-damascene Isoamyl butyrate	Dihydrojasmone lactone Para-anisaldehyde Acetoin Fruity ketal Vanillin

**Table 2:** Immature PASMC demonstrated increased susceptibility to flavored solutions by calculated LD50s. Cell death documented by lactate dehydrogenase (LDH) assay at variable dosing was used to calculate concentration at which 50% cell death would occur (lethal dose or LD50). Lower LD50s were noted for fetal and neonatal cells consistent with increased susceptibility.

	Fetal	Neonatal	Adult
Menthol	0.62	0.59	1.62
Strawberry	1.14	1.34	2.15
Tobacco	2.23	2.95	5.60
Vanilla	>10	6.61	>10
PG	>10	>10	>10
VG	>10	7.19	>10

## Supplementary Materials

The following are available online at https://www.mdpi.com/1660-4601/16/19/3635/s1, Table S1: Flavoring chemicals identified by non-targeted GCMS analysis. Flavoring chemicals present in the four refill solutions used in the study. M = menthol, S = strawberry, V = vanilla, T = tobacco.

## References

[1] Mangan D. (2013). E-cigarette sales are smoking hot, set to hit $1.7 billion. CNBC Health and Science.

[2] Rowell T.R., Tarran R. (2015). Will chronic e-cigarette use cause lung disease?. Am. J. Phys. Lung Cell Mol. Phys..

[3] King B.A., Patel R., Nguyen K.H., Dube S.R. (2015). Trends in awareness and use of electronic cigarettes among US adults, 2010–2013. Nicotine Tob. Res..

[4] Zhu S.H., Gamst A., Lee M., Cummins S., Yin L., Zoref L. (2013). The use and perception of electronic cigarettes and snus among the U.S. population. PLoS ONE.

[5] Nguyen K.H., Tong V.T., Marynak K.L., King B.A. (2016). US Adults’ Perceptions of the Harmful Effects During Pregnancy of Using Electronic Vapor Products Versus Smoking Cigarettes, Styles Survey, 2015. Prev. Chronic. Dis..

[6] Kahr M.K., Padgett S., Shope C.D., Griffin E.N., Xie S.S., Gonzalez P.J., Levison J., Mastrobattista J., Abramovici A.R., Northrup T.F. (2015). A qualitative assessment of the perceived risks of electronic cigarette and hookah use in pregnancy. BMC Public Health.

[7] Wagner N.J., Camerota M., Propper C. (2017). Prevalence and Perceptions of Electronic Cigarette Use during Pregnancy. Matern. Child Health J..

[8] Bhandari N.R., Day K.D., Payakachat N., Franks A.M., McCain K.R., Ragland D. (2018). Use and Risk Perception of Electronic Nicotine Delivery Systems and Tobacco in Pregnancy. Womens Health Issues.

[9] Oncken C., Ricci K.A., Kuo C.L., Dornelas E., Kranzler H.R., Sankey H.Z. (2017). Correlates of Electronic Cigarettes Use Before and During Pregnancy. Nicotine Tob. Res..

[10] Waldum H.L., Nilsen O.G., Nilsen T., Rorvik H., Syversen V., Sanvik A.K., Haugen O.A., Torp S.H., Brenna E. (1996). Long-term effects of inhaled nicotine. Life Sci..

[11] Husari A., Shihadeh A., Talih S., Hashem Y., El Sabban M., Zaatari G. (2016). Acute Exposure to Electronic and Combustible Cigarette Aerosols: Effects in an Animal Model and in Human Alveolar Cells. Nicotine Tob. Res..

[12] England L.J., Bunnell R.E., Pechacek T.F., Tong V.T., McAfee T.A. (2015). Nicotine and the Developing Human: A Neglected Element in the Electronic Cigarette Debate. Am. J. Prev. Med..

[13] McGrath-Morrow S.A., Hayashi M., Aherrera A., Lopez A., Malinina A., Collaco J.M., Neptune E., Klein J.D., Winickoff J.P., Breysse P. (2015). The effects of electronic cigarette emissions on systemic cotinine levels, weight and postnatal lung growth in neonatal mice. PLoS ONE.

[14] Zelikoff J.T., Parmalee N.L., Corbett K., Gordon T., Klein C.B., Aschner M. (2018). Microglia Activation and Gene Expression Alteration of Neurotrophins in the Hippocampus Following Early-Life Exposure to E-Cigarette Aerosols in a Murine Model. Toxicol. Sci..

[15] Maritz G.S., Rayise S.S. (2011). Effect of maternal nicotine exposure on neonatal rat lung development: Protective effect of maternal ascorbic acid supplementation. Exp. Lung Res..

[16] Maritz G.S., Thomas R.A. (1994). The influence of maternal nicotine exposure on the interalveolar septal status of neonatal rat lung. Cell Biol. Int..

[17] Maritz G.S., Dennis H. (1998). Maternal nicotine exposure during gestation and lactation interferes with alveolar development in the neonatal lung. Reprod. Fertil. Dev..

[18] Petre M.A., Petrik J., Ellis R., Inman M.D., Holloway A.C., Labiris N.R. (2011). Fetal and neonatal exposure to nicotine disrupts postnatal lung development in rats: Role of VEGF and its receptors. Int. J. Toxicol..

[19] Abdel-Rahman A., Dechkovskaia A.M., Sutton J.M., Chen W.C., Guan X., Khan W.A., Abou-Donia M.B. (2005). Maternal exposure of rats to nicotine via infusion during gestation produces neurobehavioral deficits and elevated expression of glial fibrillary acidic protein in the cerebellum and CA1 subfield in the offspring at puberty. Toxicology.

[20] Pauly J.R., Sparks J.A., Hauser K.F., Pauly T.H. (2004). In utero nicotine exposure causes persistent, gender-dependant changes in locomotor activity and sensitivity to nicotine in C57Bl/6 mice. Int. J. Dev. Neurosci..

[21] Lee H., Chung S., Noh J. (2016). Maternal Nicotine Exposure During Late Gestation and Lactation Increases Anxiety-Like and Impulsive Decision-Making Behavior in Adolescent Offspring of Rat. Toxicol. Res..

[22] Bahl V., Lin S., Xu N., Davis B., Wang Y.H., Talbot P. (2012). Comparison of electronic cigarette refill fluid cytotoxicity using embryonic and adult models. Reprod. Toxicol..

[23] Berkelhamer S.K., Farrow K.N. (2014). Developmental regulation of antioxidant enzymes and their impact on neonatal lung disease. Antioxid. Redox Signal..

[24] Melville J.M., Moss T.J. (2013). The immune consequences of preterm birth. Front. Neurosci..

[25] Bhandari V. (2002). Developmental differences in the role of interleukins in hyperoxic lung injury in animal models. Front. Biosci..

[26] Berkelhamer S.K., Kim G.A., Radder J.E., Wedgwood S., Czech L., Steinhorn R.H., Schumacker P.T. (2013). Developmental differences in hyperoxia-induced oxidative stress and cellular responses in the murine lung. Free Radic. Biol. Med..

[27] Datta A., Kim G.A., Taylor J.M., Gugino S.F., Farrow K.N., Schumacker P.T., Berkelhamer S.K. (2015). Mouse lung development and NOX1 induction during hyperoxia are developmentally regulated and mitochondrial ROS dependent. Am. J. Phys. Lung Cell Mol. Phys..

[28] Makri A., Goveia M., Balbus J., Parkin R. (2004). Children’s susceptibility to chemicals: A review by developmental stage. J. Toxicol. Environ. Health B Crit Rev..

[29] Lambers D.S., Clark K.E. (1996). The maternal and fetal physiologic effects of nicotine. Semin. Perinatol..

[30] Leigh N.J., Lawton R.I., Hershberger P.A., Goniewicz M.L. (2016). Flavourings significantly affect inhalation toxicity of aerosol generated from electronic nicotine delivery systems (ENDS). Tob. Control..

[31] Gerloff J., Sundar I.K., Freter R., Sekera E.R., Friedman A.E., Robinson R., Pagano T., Rahman I. (2017). Inflammatory Response and Barrier Dysfunction by Different e-Cigarette Flavoring Chemicals Identified by Gas Chromatography-Mass Spectrometry in e-Liquids and e-Vapors on Human Lung Epithelial Cells and Fibroblasts. Appl. In Vitro Toxicol..

[32] Lerner C.A., Sundar I.K., Yao H., Gerloff J., Ossip D.J., McIntosh S., Robinson R., Rahman I. (2015). Vapors produced by electronic cigarettes and e-juices with flavorings induce toxicity, oxidative stress, and inflammatory response in lung epithelial cells and in mouse lung. PLoS ONE.

[33] Tierney P.A., Karpinski C.D., Brown J.E., Luo W., Pankow J.F. (2016). Flavour chemicals in electronic cigarette fluids. Tob. Control..

[34] Wang L., Zhan Y., Li Q., Zeng D.D., Leischow S.J., Okamoto J. (2015). An Examination of Electronic Cigarette Content on Social Media: Analysis of E-Cigarette Flavor Content on Reddit. Int. J. Environ. Res. Public Health.

[35] Czogala J., Goniewicz M.L., Fidelus B., Zielinska-Danch W., Travers M.J., Sobczak A. (2014). Secondhand exposure to vapors from electronic cigarettes. Nicotine Tob. Res..

[36] Wang T.W., Marynak K.L., Agaku I.T., King B.A. (2017). Secondhand Exposure to Electronic Cigarette Aerosol Among US Youths. JAMA Pediatr..

[37] Johnson J.M., Naeher L.P., Yu X., Rathbun S.L., Muilenburg J.L., Wang J.S. (2018). Air monitoring at large public electronic cigarette events. Int. J. Hyg. Environ. Health.

[38] Hutzler C., Paschke M., Kruschinski S., Henkler F., Hahn J., Luch A. (2014). Chemical hazards present in liquids and vapors of electronic cigarettes. Arch. Toxicol..

[39] Zahedi A., Phandthong R., Chaili A., Leung S., Omaiye E., Talbot P. (2019). Mitochondrial Stress Response in Neural Stem Cells Exposed to Electronic Cigarettes. Science.

[40] Omaiye E.E., McWhirter K.J., Luo W., Pankow J.F., Talbot P. (2019). High-Nicotine Electronic Cigarette Products: Toxicity of JUUL Fluids and Aerosols Correlates Strongly with Nicotine and Some Flavor Chemical Concentrations. Chem. Res. Toxicol..

[41] Hua M., Omaiye E.E., Luo W., McWhirter K.J., Pankow J.F., Talbot P. (2019). Identification of Cytotoxic Flavor Chemicals in Top-Selling Electronic Cigarette Refill Fluids. Sci. Rep..

[42] Omaiye E.E., McWhirter K.J., Luo W., Tierney P.A., Pankow J.F., Talbot P. (2019). High concentrations of flavor chemicals are present in electronic cigarette refill fluids. Sci. Rep..

[43] Zare S., Nemati M., Zheng Y. (2018). A systematic review of consumer preference for e-cigarette attributes: Flavor, nicotine strength, and type. PLoS ONE.

[44] Farrow K.N., Groh B.S., Schumacker P.T., Lakshminrusimha S., Czech L., Gugino S.F., Russell J.A., Steinhorn R.H. (2008). Hyperoxia increases phosphodiesterase 5 expression and activity in ovine fetal pulmonary artery smooth muscle cells. Circ. Res..

[45] Lakshminrusimha S., Steinhorn R.H. (1999). Pulmonary vascular biology during neonatal transition. Clin. Perinatol..

[46] Hanouni M., Bernal G., McBride S., Narvaez V.R., Ibe B.O. (2016). Hypoxia and hyperoxia potentiate PAF receptor-mediated effects in newborn ovine pulmonary arterial smooth muscle cells: Significance in oxygen therapy of PPHN. Physiol. Rep..

[47] Black S.M., Field-Ridley A., Sharma S., Kumar S., Keller R.L., Kameny R., Maltepe E., Datar S.A., Fineman J.R. (2017). Altered Carnitine Homeostasis in Children With Increased Pulmonary Blood Flow Due to Ventricular Septal Defects. Pediatr. Crit. Care Med..

[48] Lakshminrusimha S., Swartz D.D., Gugino S.F., Ma C.X., Wynn K.A., Ryan R.M., Russell J.A., Steinhorn R.H. (2009). Oxygen concentration and pulmonary hemodynamics in newborn lambs with pulmonary hypertension. Pediatr. Res..

[49] Fineman J.R., Wong J., Soifer S.J. (1993). Hyperoxia and alkalosis produce pulmonary vasodilation independent of endothelium-derived nitric oxide in newborn lambs. Pediatr. Res..

[50] Lakshminrusimha S., Russell J.A., Steinhorn R.H., Ryan R.M., Gugino S.F., Morin F.C., Swartz D.D., Kumar V.H. (2006). Pulmonary arterial contractility in neonatal lambs increases with 100% oxygen resuscitation. Pediatr. Res..

[51] Zayek M., Cleveland D., Morin F.C. (1993). Treatment of persistent pulmonary hypertension in the newborn lamb by inhaled nitric oxide. J. Pediatr..

[52] Pearl J.M., Nelson D.P., Raake J.L., Manning P.B., Schwartz S.M., Koons L., Shanley T.P., Wong H.R., Duffy J.Y. (2002). Inhaled nitric oxide increases endothelin-1 levels: A potential cause of rebound pulmonary hypertension. Crit. Care Med..

[53] McMullan D.M., Bekker J.M., Johengen M.J., Hendricks-Munoz K., Gerrets R., Black S.M., Fineman J.R. (2001). Inhaled nitric oxide-induced rebound pulmonary hypertension: Role for endothelin-1. Am. J. Physiol. Heart Circ. Physiol..

[54] Thebaud B., Abman S.H. (2007). Bronchopulmonary dysplasia: Where have all the vessels gone? Roles of angiogenic growth factors in chronic lung disease. Am. J. Respir. Crit. Care Med..

[55] Misra M., Leverette R.D., Cooper B.T., Bennett M.B., Brown S.E. (2014). Comparative in vitro toxicity profile of electronic and tobacco cigarettes, smokeless tobacco and nicotine replacement therapy products: E-liquids, extracts and collected aerosols. Int. J. Environ. Res. Public Health.

[56] Rowell T.R., Reeber S.L., Lee S.L., Harris R.A., Nethery R.C., Herring A.H., Glish G.L., Tarran R. (2017). Flavored e-cigarette liquids reduce proliferation and viability in the CALU3 airway epithelial cell line. Am. J. Phys. Lung Cell Mol. Phys..

[57] Ito S., Kume H., Shiraki A., Kondo M., Makino Y., Kamiya K., Hasegawa Y. (2008). Inhibition by the cold receptor agonists menthol and icilin of airway smooth muscle contraction. Pulm. Pharmacol. Ther..

[58] Wang H.W., Liu S.C., Chao P.Z., Lee F.P. (2016). Menthol inhibiting parasympathetic function of tracheal smooth muscle. Int. J. Med. Sci..

[59] Bitzer Z.T., Goel R., Reilly S.M., Elias R.J., Silakov A., Foulds J., Muscat J., Richie J.P. (2018). Effect of flavoring chemicals on free radical formation in electronic cigarette aerosols. Free Radic. Biol. Med..

[60] Budinger G.R., Mutlu G.M., Urich D., Soberanes S., Buccellato L.J., Hawkins K., Chiarella S.E., Radigan K.A., Eisenbart J., Agrawal H. (2011). Epithelial cell death is an important contributor to oxidant-mediated acute lung injury. Am. J. Respir. Crit. Care Med..

[61] Korsmeyer S.J., Yin X.M., Oltvai Z.N., Veis-Novack D.J., Linette G.P. (1995). Reactive oxygen species and the regulation of cell death by the Bcl-2 gene family. Biochim. Biophys. Acta.

[62] Kjaeve J., Ingebrigtsen T., Naess L., Bjertnaes L., Vaage J. (1996). Methylprednisolone attenuates airway and vascular responses induced by reactive oxygen species in isolated, plasma-perfused rat lungs. Free Radic. Res..

[63] National Cancer Institute Tobacco Control Research (2009). Research Topic: Menthol and Tobacco.

[64] Villanti A.C., Collins L.K., Niaura R.S., Gagosian S.Y., Abrams D.B. (2017). Menthol cigarettes and the public health standard: A systematic review. BMC Public Health.

[65] Park S.J., Foreman M.G., Demeo D.L., Bhatt S.P., Hansel N.N., Wise R.A., Soler X., Bowler R.P. (2015). Menthol cigarette smoking in the COPDGene cohort: Relationship with COPD, comorbidities and CT metrics. Respirology.

[66] Famele M., Ferranti C., Abenavoli C., Palleschi L., Mancinelli R., Draisci R. (2015). The chemical components of electronic cigarette cartridges and refill fluids: Review of analytical methods. Nicotine Tob. Res..

[67] Behar R.Z., Luo W., McWhirter K.J., Pankow J.F., Talbot P. (2018). Analytical and toxicological evaluation of flavor chemicals in electronic cigarette refill fluids. Sci. Rep..

[68] Shields P.G., Berman M., Brasky T.M., Freudenheim J.L., Mathe E., McElroy J.P., Song M.A., Wewers M.D. (2017). A Review of Pulmonary Toxicity of Electronic Cigarettes in the Context of Smoking: A Focus on Inflammation. Cancer Epidemiol. Biomarkers Prev..

[69] Behrsing H., Hill E., Raabe H., Tice R., Fitzpatrick S., Devlin R., Pinkerton K., Oberdorster G., Wright C., Wieczorek R. (2017). In vitro exposure systems and dosimetry assessment tools for inhaled tobacco products: Workshop proceedings, conclusions and paths forward for in vitro model use. Altern. Lab. Anim..

[70] Zhu S.H., Sun J.Y., Bonnevie E., Cummins S.E., Gamst A., Yin L., Lee M. (2014). Four hundred and sixty brands of e-cigarettes and counting: Implications for product regulation. Tob. Control..

[71] Sosnowski T.R., Kramek-Romanowska K. (2016). Predicted Deposition of E-Cigarette Aerosol in the Human Lungs. J. Aerosol. Med. Pulm. Drug Deliv..

