# Supplementary material for: In Vitro Consequences of Electronic-Cigarette Flavoring Exposure on the Immature Lung
Source: Int J Environ Res Public Health. 2019 Sep 27;16(19):3635. doi: 10.3390/ijerph16193635 (PMC6801380; doi:10.3390/ijerph16193635)
Supplement: Supplementary file 1 [file ijerph-16-03635-s001.pdf]

[illegible]		[illegible]	[illegible]	
carvone	1,8-cineole	propylene glycol diacetate	dihydrojasmone lactone	vanillin propylene glycol acetal
alpha-terpineol	laevo-carveol	methyl cinnamate	para-anisaldehyde	ethyl vanillin
(±)-menthol	butyl alcohol	delta-decalactone	methyl propionate	gamma-hexalactone
(±)-menthone	trans-carveol	(Z)-linalool oxide (furanoid)	sulfurol	acetyl propionyl
(-)-menthone	dipentene	(Z)-3-hexen-1-ol	ethyl vanillin propylene glycol acetal	vanillin
phenethyl alcohol	beta-pinene	2-methyl butyric acid	levulinic acid	ortho-guaiacol
pulegone	cis-dihydrocarvone	methyl sulfone	heliotropin	propionic acid
(-)-isopulegol	alpha-pinene	methyl (E)-cinnamate	para-anisyl alcohol	para-dimethyl hydroquinone
isopulegol	ethyl phenyl acetate	isovaleraldehyde propylene glycol acetal	gamma-nonalactone	isoamyl isovalerate
(R)-(+)-pulegone	anethol	benzaldehyde	delta-octalactone	isobutyl acetate
(±)-isomenthone	(E)-anethol	(E)-linalool oxide (furanoid)	acetoin	2-methyl-3-heptanone
3-octanol	cinnamyl acetate	hexanoic acid	fruity ketal	gamma-octalactone
cis-carveol	trans-dihydrocarvone	2-butanol	dihydrojasmone lactone	(E,E)-2,6-nonadienal
gamma-undecalactone	laevo-beta-pinene	ethyl acetate		
(E)-3-hexen-1-ol	(E)-cinnamyl acetate	ethyl 2-methyl butyrate	**TOBACCO**	
2-ethyl-1-hexanol	nonisyl acetate	isoamyl acetate	beta-damascone	
menthyl acetate racemic	menthyl acetate	isovaleraldehyde	ethyl cyclopentenolone	
		(Z)-3-hexen-1-yl acetate	isoamyl butyrate	
		acetone		
		allyl hexanoate		

[illegible]
benzaldehyde propylene glycol acetal

M+V: leerall, dihydrocarveol

S+V: butyric acid, gamma-decalactone, acetic acid, maltol, isoamyl propionate, 2,3-butane diol, 3-pentanol, 4-heptanol, ethyl butyrate

S+T: dihydroxyacetone

M+V+T: cyclotene

M+S+T: (E)-2-pentenal

M+S+V+T: benzophenone, hydroxyacetone, ethyl maltol
